# Supplementary material for: Targeting Metabolic Vulnerabilities to Overcome Prostate Cancer Resistance: Dual Therapy with Apalutamide and Complex I Inhibition
Source: Cancers (Basel). 2023 Nov 28;15(23):5612. doi: 10.3390/cancers15235612 (PMC10705744; doi:10.3390/cancers15235612)
Supplement: Supplementary file 1 [file cancers-15-05612-s001.zip › cancers-2640837-supplementary.pdf]

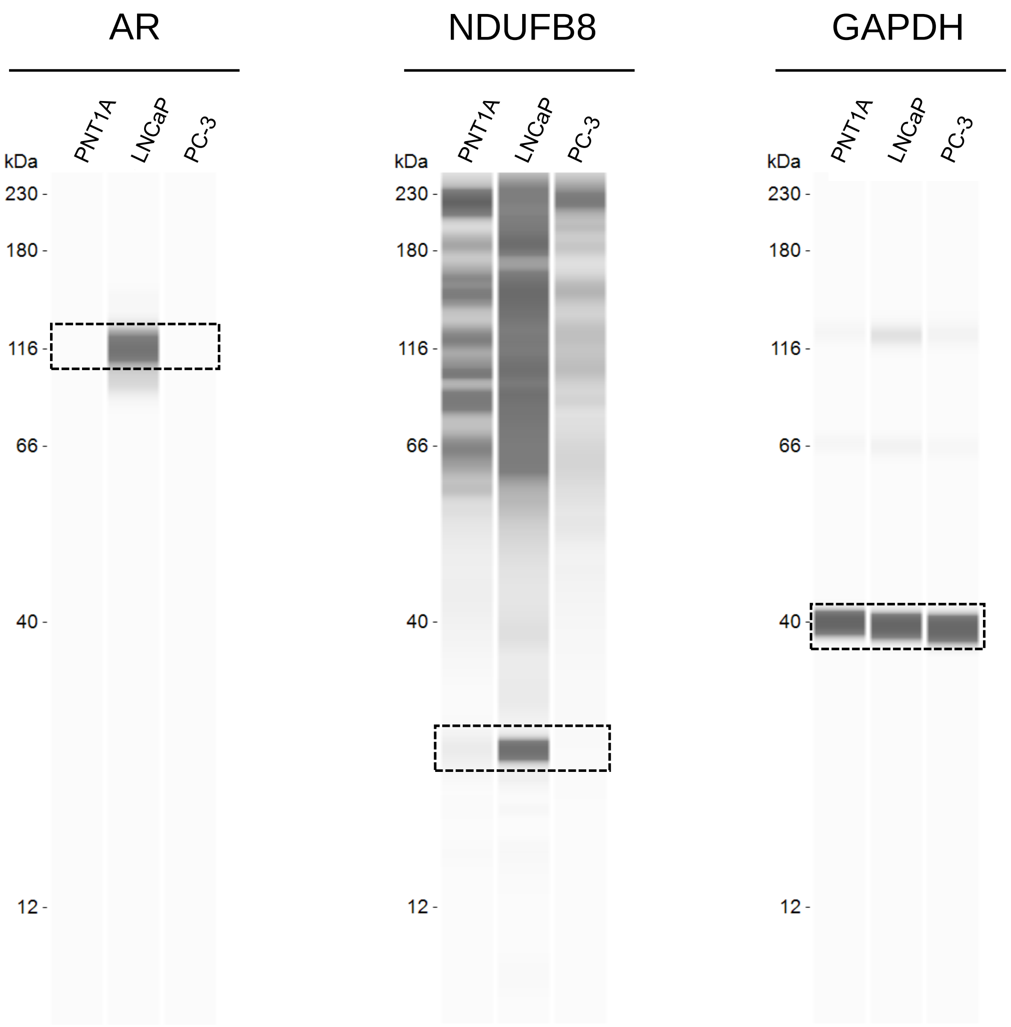

Figure S1. Original images of western blots. Molecular weight markers and protein lanes of AR, NDUFB8 and GAPDH are shown for Figure 1D.

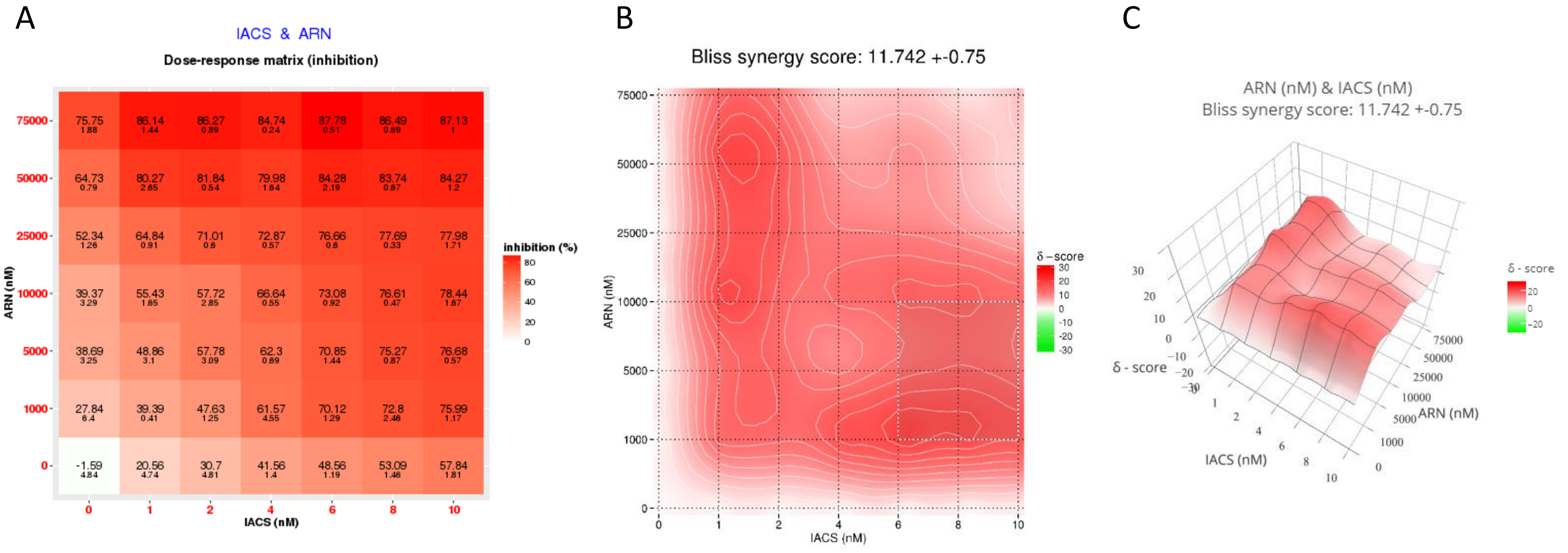

Figure S2. Synergy analysis of ARN and IACS in LNCaP cells. (**A**) Heat map showing inhibition effect (%) of different ARN and IACS concentrations. (**B**) Positive values are considered synergistic (red) and are shown in a 2D and (**C**) 3D-plot.

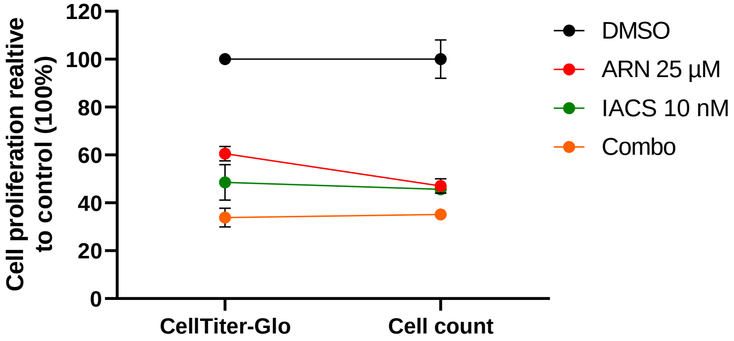

Figure S3. Comparison of cell proliferation by CellTiter-Glo 2.0 and cell counting in LNCaP cells. Cell counting was performed by Hoechst 33342 staining. Stained nuclei were quantified by ImageJ software. N CellTiter-Glo = 9, N cell count= 6.
